# Recent nanoengineered therapeutic advancements in sepsis management

**DOI:** 10.3389/fbioe.2024.1495277

**Published:** 2024-12-05

**Authors:** Li Liu, Li Li, Ting Wang, Zheyu Li, Bingpeng Yan, Ruirong Tan, Anqi Zeng, Wenbo Ma, Xin Zhu, Zhujun Yin, Chunhua Ma

**Affiliations:** ^1^ Translational Chinese Medicine Key Laboratory of Sichuan Province, Sichuan Institute for Translational Chinese Medicine, Sichuan Academy of Chinese Medicine Sciences, Chengdu, China; ^2^ Antibiotics Research and Re-evaluation Key Laboratory of Sichuan Province, Sichuan Industrial Institute of Antibiotics, Chengdu University, Chengdu, China; ^3^ State Key Laboratory of Emerging Infectious Diseases, Carol Yu Centre for Infection, Department of Microbiology, School of Clinical Medicine, Li Ka Shing Faculty of Medicine, The University of Hong Kong, Hong Kong, China; ^4^ Hunan Provincial Key Laboratory of the Research and Development of Novel Pharmaceutical Preparations, The “Double-First Class” Application Characteristic Discipline of Hunan Province (Pharmaceutical Science), Changsha Medical University, Changsha, China; ^5^ State Key Laboratory of Trauma, Burns and Combined Injury, Department of Shock and Transfusion, Research Institute of Surgery, Daping Hospital, Army Medical University, Chongqing, China

**Keywords:** sepsis, nanomaterials, drug delivery, safety, antibiotic, antimicrobial peptides, biomimetic nanoparticles, immunomodulatory

## Abstract

Sepsis (defined as sepsis 3.0) is a life-threatening organ dysfunction caused by a dysregulated host response to a variety of pathogenic microorganisms. Characterized by high morbidity and mortality, sepsis has become a global public health problem. However, there is a lack of appropriate diagnostic and therapeutic strategies for sepsis and current management rely on the limited treatment strategies. Recently, nanomedicines targeting and controlling the release of bio-active agents have shown excellent potency in sepsis management, with improved therapeutic efficacy and reduced adverse effects. In this review, we have summarized the advantages of nanomaterials. Also, the preparation and efficacy of the main categories of anti-sepsis nanomedicines applied in sepsis management are described in detail, including antibiotic-coated nanomaterials, antimicrobial peptides-coated nanomaterials, biomimetic nanomaterials, nanomaterials targeting macrophages and natural products loaded nanomaterials. These advances in nanomedicines establish the huge potential for nanomaterials-based sepsis management, especially in the improved pharmaceutical and pharmacological properties, enhanced therapeutic efficacy, controllable drug-targeting and reduced side effects. To further facilitate clinical translation of anti-sepsis nanomedicines, we propose that the issues involving safety, regulatory laws and cost-effectiveness should receive much more attention in the future.

## 1 Introduction

Sepsis is defined as a clinical syndrome characterized by physiological, pathological, and biochemical abnormalities induced by an invading pathogen, causing dysregulated host immune response and resulting as ultimately responsible for life-threatening organ dysfunction (Deutschman and Tracey, 2014; Singer et al., 2016; Rhee et al., 2019). A recent Global Burden of Diseases Report showed that approximately 50 million people worldwide suffered from sepsis each year, among which more than 20% patient died, and that sepsis was the leading cause of death worldwide (Vincent et al., 2014; Rudd et al., 2020). In addition, once suffered septic shock and multiple organ failures, the expensive medical costs follows (Fleischmann et al., 2016; Vincent et al., 2014).

Over the last few decades, with the comprehensive understanding and application of anti-sepsis therapies, the mortality rate has been reduced dramatically, including timely anti-infective therapy, adequate fluid resuscitation, and vasoactive drug therapy, *etc.* However, there are non-negligible shortcomings in conventional anti-sepsis therapies, such as poor bioavailability, lack of specific targeting, multi-drug resistance (MDR) and side effects (Wang Y. et al., 2023). Notably, therapies with low targeting have a huge potential for systemic toxicity. Usually, broad-spectrum antibiotics show an excellent therapeutic effect in infective sepsis, however, drug resistance in pathogens can adversely affect sepsis and can double the mortality rate (Soni et al., 2022). As estimated, MDR to pathogenic pathogens might be responsible for approximately 215,000 deaths in neonatal sepsis. Therefore, facing the significant clinical need for specific therapeutic options, it is urgent to develop innovative medications and diagnostic methods for sepsis management.

Over the years, nanotechnology has revolutionized the traditional pharmaceutical industry, bringing new prospects and alternative options for the research and development of novel diagnostic and therapeutic strategies. Specifically, the controllable physicochemical features of nanomaterials, such as size, shape, and surface properties, have significantly increased stability and bioavailability (Guo et al., 2021). Furthermore, the surfaces of nanomaterials can be diversely functionalized, which can greatly improve their capacity for targeted delivery to specific cells or tissues. These advantages have prompted research into the development of promising immunomodulatory nanosystems that facilitate the adjunctive therapy of sepsis and other immunological disorders (Feng et al., 2019; Lasola et al., 2020). Despite these advantages, certain non-negligible issues hinder the progression and translation of anti-sepsis nanomedicines into clinical practice, including safety concerns, regulatory laws and cost-benefit considerations related to nanomaterials (Chu et al., 2023).

**FIGURE 1:**
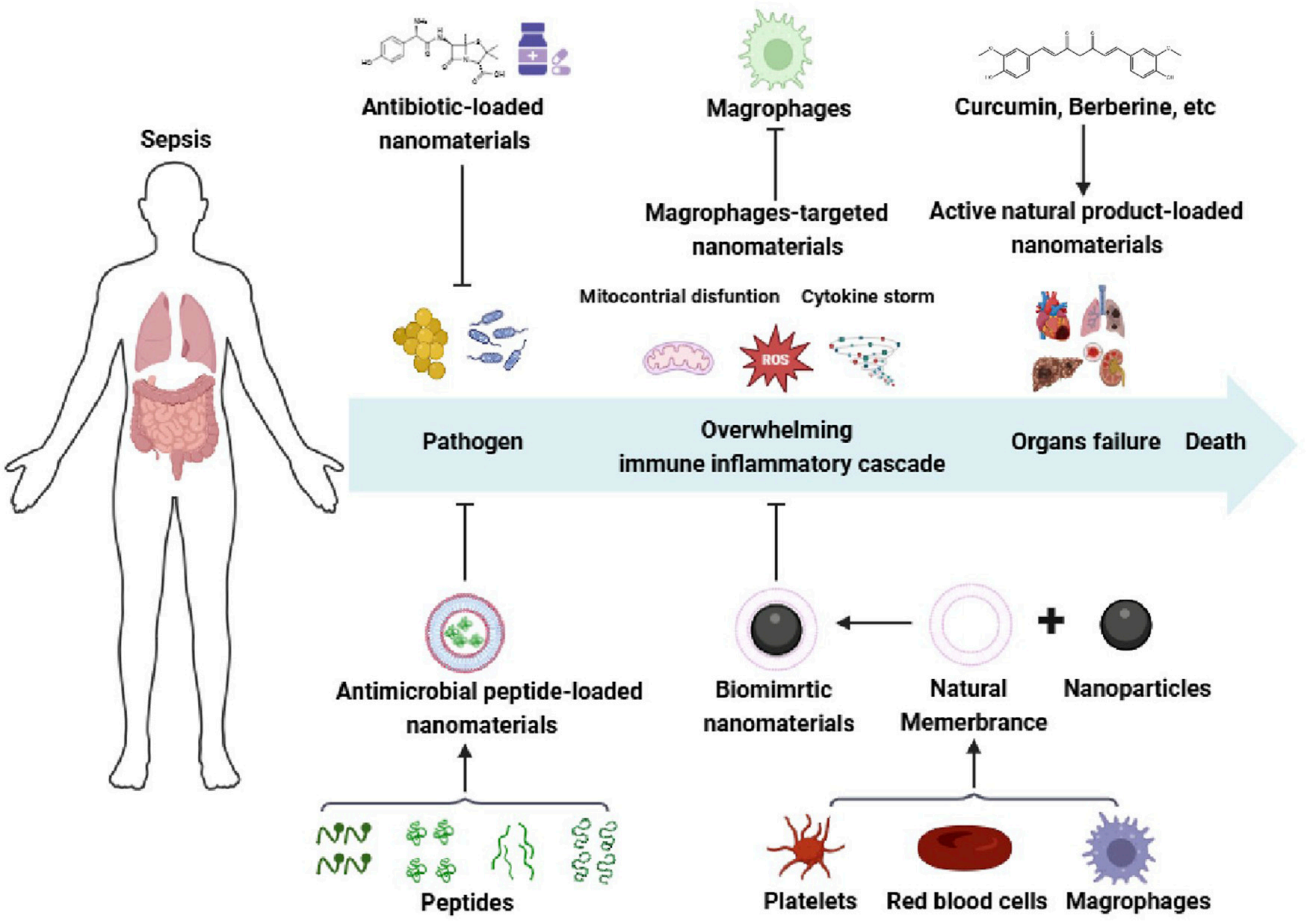
Schematic illustration of the sepsis pathophysiological events occurring and nanomedicines for sepsis. Created with BioRender.com.

In this review, we aim to highlight the significant potential of nanomedicines in sepsis management (Figure 1), including antibiotic-coated nanomaterials, antimicrobial peptides-coated nanomaterials, biomimetic nanomaterials, nanomaterials targeting macrophage and natural products loaded nanomaterials. It also emphasizes the safety issues and regulatory challenges to facilitate clinical translation of anti-sepsis nanomedicines.

## 2 Pathophysiology of sepsis

Sepsis has been defined as life-threatening organ dysfunction resulting from the unbalanced host immune against infection, which was recognized as sepsis 3.0 firstly proposed by the international guidelines in 2016 (Townsend et al., 2016; Rhee and Klompas, 2017; Fu et al., 2024). Given the clinical heterogeneity of sepsis, multiple pathophysiological mechanisms are involved in sepsis (Figure 1), including coagulation dysfunction, overwhelming immune inflammatory cascade, endothelial capillary leakage, *etc.* Triggered by an excessive inflammation response, longer-term immunosuppression of the septic patients is responsible for the tissue damage, eventually leading to the failure of multiple organs. In response to pathogens and toxic components released by pathogens and host, the innate immune cells initiate an inflammatory response to release inflammatory mediators by recognizing pathogen-associated molecular patterns (PAMPs) or damage-associated molecular patterns (DAMPs), further recruiting vascular immune cells to the sites of infection, such as neutrophils, monocytes and macrophages (Vasconcelos et al., 2016; Greene et al., 2022; Zindel and Kubes, 2020). Usually, the inflammatory responses are resolved via the production of anti-inflammatory cytokines, antibacterial peptide, and stress hormones to recover the host immune system to homeostasis (Choudhary, 2022; Yuk et al., 2018). Conversely, with residual pathogenic microorganisms, an over-activation of the immunoinflammatory cascade (also known as“cytokine storm”) bursts, which is characterized by an overwhelming release of pro-inflammatory cytokines and mediators, including tumor necrosis factor-α (TNF-α), interleukin-1β (IL-1β), IL-6, IL-8, reactive oxygen species (ROS), nitric oxide and matrix metalloproteinases (Netea et al., 2017; Cao et al., 2019). In the post-immune stage, the dysregulated host immune induced by the exhaustion of immune cells facilitates to persistent immunosuppression, ultimately resulting in damage to multiple organs (lungs, liver, kidneys, heart), and increased late-period mortality (Netea et al., 2017). Therefore, it is a generally acknowledged fact that the intensity and duration of immune-inflammatory cascade decides the mortality and outcome of sepsis.

Sepsis is triggered by the event of the recognition of PAMPs, which include bacterial DNA, small RNA (sRNA), double-stranded RNA (dsRNA) and membrane-forming molecules such as lipopolysaccharides (LPS) (Wang X. et al., 2023). Once detecting the attacks by pathogenic pathogens, a series of complex molecular and cellular immune response activate to combat the invading pathogens, which share group of PAMPs in the body. Subsequently, the antigen-presenting cells (APCs) of the host, including monocyte-macrophages, dendritic cells, B cells, Langerhans cells, *etc.*, recognize PAMPs via pattern-recognition receptors and present them to immune cells (Cao et al., 2019), initiating a “precise strike” to fight against the infection.

Patients with sepsis develop an excessive inflammatory response in the early stage, followed by a prolonged period of immunosuppression and catabolism clinical syndrome (Gentile et al., 2012; Nakamura et al., 2022). Currently, the main features of this clinical syndrome are markedly increased of C-reactive protein (CRP) concentrations, neutrophilia, and the release of immature myeloid cells (Hawkins et al., 2018). Immature myeloid cells have defective antimicrobial activity and produce anti-inflammatory cytokines when mobilized to circulation, downregulate the inflammation and resulting in functional immunosuppression.

With an ever-deepening understanding of pathogenesis of sepsis, intracellular organelle damage and a variety of abnormal molecular events are involved in the development of sepsis, such as mitochondrial dysfunction (Hu et al., 2024; Kumar et al., 2024), endoplasmic reticulum stress (Gong et al., 2022), multiple forms of cell death (apoptosis, pyroptosis, necrosis, ferroptosis and cuproptosis), coagulation abnormalities and more, eventually leading to multiple organs failure (Zhang et al., 2023; Li et al., 2023). Based on the after mentioned pathogenesis of sepsis, several antiseptic medications were developed and widely applied, including of antibiotics (Yang et al., 2021; Roth et al., 2023), antibacterial peptide (Ma et al., 2022), immunomodulatory and anti-inflammatory medications (Cao et al., 2022), *etc.* Significantly, potential risks and safety for sepsis have attracted much more attention worldwide, including resistance of antibiotic, a sudden onset of immunosuppression and poor prognosis. Thus, discovering the anti-septic drug delivery systems with safer, more efficient and targeted potency have been recognized as the novel strategies to combat pathogenic microorganisms and catastrophic immunosuppression in the septic patients.

## 3 An overview of nanotechnology

Nanotechnology is a highly interdisciplinary field that integrates physics, surface chemistry, materials science, biology, pharmaceutics, electronics, computer science, *etc* (Vasconcelos and Santos, 2023). It has been pivotal in the design and application of nanosystems, addressing challenges in pharmaceutical and medical sciences. These innovative nanomedicines offer enhanced stability, specificity, sensitivity, and quality control, ensuring more effective clinical therapeutic outcomes compared to traditional approaches. Nanomaterials, which are materials with dimensions between 1 and 100 nm, exhibit unique chemical, physical, and surface properties (Vasconcelos and Santos, 2023; Makabenta et al., 2021; Sindhwani and Chan, 2021). Minor alterations in their molecular composition can significantly alter their size, shape, and charge, leading to improved circulation times and distinct biodistribution profiles compared to unmodified nanomaterials (Gabizon et al., 1994; Green et al., 2006). Notably, nanomaterials can be engineered to deliver drugs or small molecules through various mechanisms such as electrostatic adsorption (Kim et al., 2024; Ho et al., 2023), hydrophobic interactions (Tang et al., 2022), entrapment (Snider et al., 2021), π-π adsorption (Xing et al., 2024; Tang et al., 2024), and covalent binding (Li et al., 2024). Respond to specific micro-environments, these multi-responsive nanomaterials-based systems exhibit reduced systemic toxicity and enhanced therapeutic efficacy (Guo et al., 2021; Guo et al., 2021; Shimanovich and Gedanken, 2016).

**FIGURE 2:**
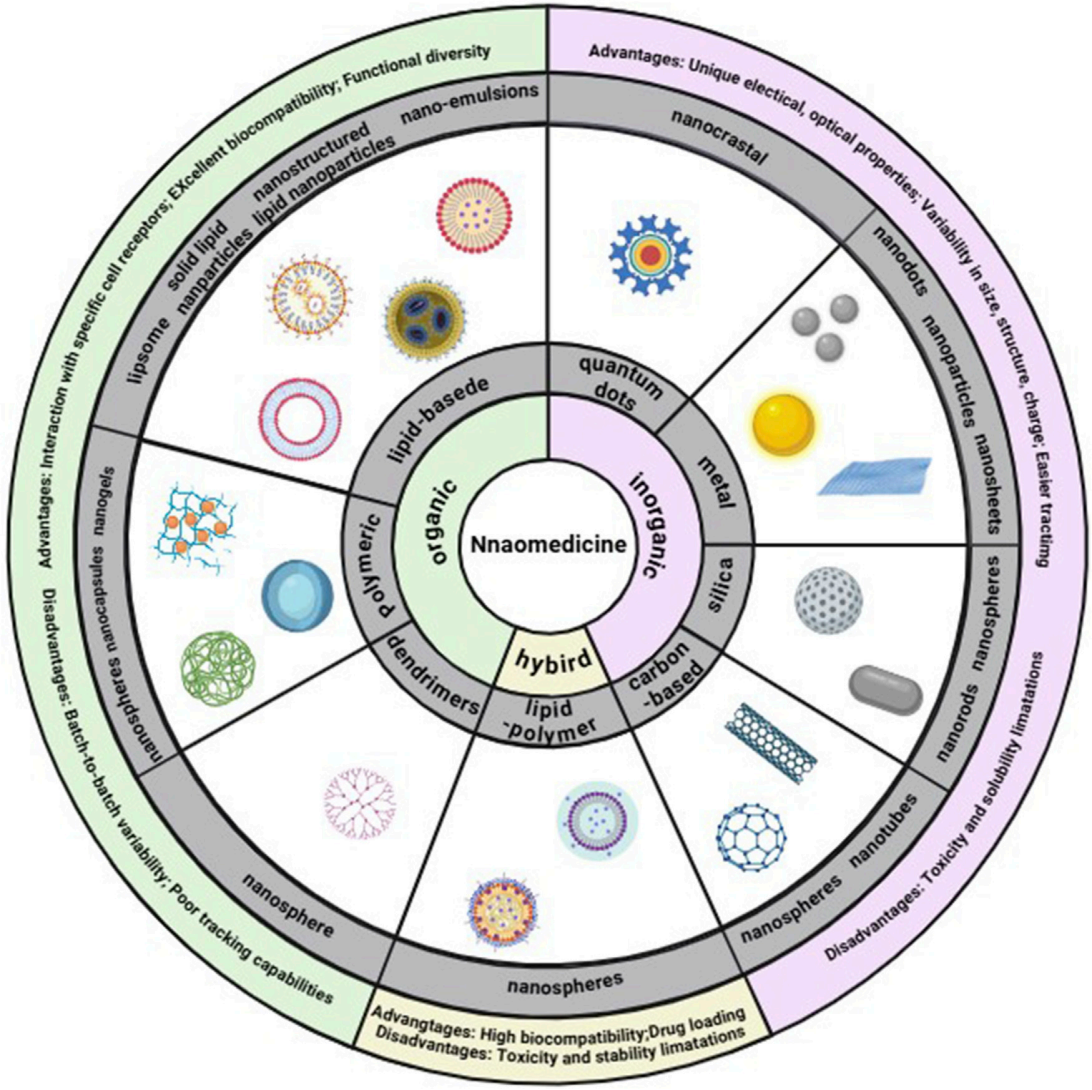
Classes of nanomaterials. Each class has numerous advantages and disadvantages. Image created with BioRender.com.

Nanomaterials are broadly categorized into organic, inorganic, and hybrid types (Figure 2). Organic nanomaterials, such as lipid-based, polymer-based, and dendrimers, are known for their excellent biocompatibility, low toxicity, and minimal immunological responses. They also have the advantage of interacting with specific receptors (Saraiva et al., 2016). Inorganic nanomaterials are typically divided into metallic, quantum dot, silica, and carbon-based materials. Metallic nanomaterials include nanoparticles (NPs), nanosheets, and nanorods, while silica and carbon-based NPs are also considered inorganic. Despite some disadvantages, such as batch variability and limited modification capabilities, inorganic nanomaterials offer advantages like precise size and shape control, and ease of surface functionalization (Saraiva et al., 2016). These materials are also more readily trackable using optical microscopes or other analytical techniques.

The benefits of nanomaterials can be encapsulated in the following points: (1) Their size diversity allows for tailored applications in treating specific diseases, with smaller nanomaterials often used for diagnostic purposes and larger ones for drug delivery or clearance (Toy et al., 2014); (2) The shape of nanomaterials influences cellular uptake rates, with rods being most efficiently internalized, followed by spheres and cubes. The shape also affects blood circulation as well as the ability to marginate and bind to other elements (Ding et al., 2023); (3) the surface charge of nanomaterials is crucial for blood circulation and initial cell membrane absorption. The presence of different surface charges on a material can facilitate the loading of drugs and therapeutic agents (Rasmussen et al., 2020); (4) Surface functionalization with targeting ligands enables selective adherence to bacterial cells (Molinaro et al., 2016), delivering drugs precisely to the infected site (Koo et al., 2017). This approach reduces off-target effects, minimizes damage to healthy tissue, decreases toxicity, and enhances the retention time and efficiency of targeted delivery (Saraiva et al., 2016).

## 4 Nanodiagnostic techniques and challenges in sepsis

### 4.1 Nanomaterials for detecting pathogens

The rapid and accurate determination of infection is crucial for the diagnosis and early treatment of sepsis, thus facilitating the prognosis of patients. There are several traditional means of pathogenic diagnosis in sepsis, including culture-based techniques, polymerase chain reaction (PCR) and second-generation sequencing technology. The primary limitations of the above mentioned methods are as follows: (1) Results are not available for several days, which can delay the start of treatment; (2) Approximately 35% of cases that are initially suspected to be septic test negative in culture tests, potentially leading to misdiagnosis; (3) These methods frequently exhibit low sensitivity and specificity, which may result in the inaccurate identification of pathogens (Salomão et al., 2019). Therefore, there is an urgent need for technologies that can not only rapidly and accurately detect infections but also reliably identify pathogens and their functional traits.

Recently, nanomaterials have shown great promise in developing more sensitive and specific diagnostic tools for early bacterial infection detection. In particular, nanomaterials have been extensively studied for their detection capabilities. Functionalized with therapeutic antibiotics, antimicrobial peptides or active compounds, NPs selectively bind to components of the bacterial cell wall, such as lipopolysaccharides (LPS), or peptidoglycan. For instance, Zhu et al. demonstrated that Au NPs modified with ethanolamine and *Escherichia coli* O111:B4 LPS could be used to detect LPS through a color change mechanism. This is achieved by the binding of aptamers (G-probes) to ethanolamine, a component found in all LPS variants, causing the Au NPs to aggregate and shift color from red to blue. Furthermore, magnetic nanoparticles (MNPs) and carbon nanotubes (CNTs) have been explored for their potential in bacterial detection, offering new avenues for rapid and accurate diagnostic methods (Zhu et al., 2019).

### 4.2 Nanobiosensors for detecting biomarkers

Septic biomarkers, including procalcitonin (PCT) (Reinhart and Meisner, 2011), C-reactive protein (CRP) (Eschborn and Weitkamp, 2019), and IL-6 (Eichberger and Resch, 2022), have been identified for clinical diagnosis and management of sepsis. However, their sensitivity and specificity are limited, underscoring the urgent need for more precise diagnostic tools. With the advantages of high speed, high sensitivity and portability, nanobiosensors are suitable for bedside rapid and simultaneous detection of these biomarkers (Alba-Patiño et al., 2022).

Conjugating nanomaterials with antibodies or peptides that target sepsis biomarkers specifically can identify and quantify sepsis biomarkers by capturing significant alterations in the nanomaterials’ electrical conductivity or fluorescence intensity via the unique binding. Nanomaterials-based chemical biosensors have shown great potential in sepsis biomarker sensing due to their distinctive properties, such as carbon nanotubes (CNTs), Au NPs (Munge et al., 2009), and MNPs. In addition, electrochemical biosensors based on nanomaterials can achieve high-sensitivity and minimal-sample-amount detection of PCT in undiluted serum, which exhibit good correlation with the conventional ELISA method, but with less time consumption.

### 4.3 Nanoparticle imaging techniques for diagnosing sepsis

Nanotechnology has risen as innovative tools for diagnosing and monitoring sepsis. Based on the principle of nuclear magnetic resonance, magnetic resonance imaging (MRI) is designed to obtain detailed images of the internal structures of human body. For example, superparamagnetic iron oxide nanoparticles (SPIONs) have been extensively researched for their use as MRI contrast agents in sepsis imaging, enhancing the visualization of affected areas. Furthermore, optical imaging, a non-invasive technique that employs light to illuminate organs and tissues, is another valuable approach. Near-infrared fluorescence (NIRF) imaging, capitalizes on the near-infrared light’s ability to penetrate biological tissues, providing superior imaging capabilities for detecting sepsis. In addition, since the cell metabolism in areas of inflammation and infection is hyperactive, positron emission tomography (PET) technology can be applied to detect sepsis-induced specific tissue injuries, the degree of inflammatory activity, assisting clinician to design the treatment strategies and evaluate patient outcomes. Radiolabeled NPs, functionalized with targeting ligands that selectively bind to sepsis biomarkers, enable targeted imaging of specific infected tissues, such as iron oxide or Au NPs. These radiolabeled NPs have been recognized as potential PET contrast agents in sepsis diagnosis. For instance, a nano-radiotracer combining neutrophil-specific biomolecule and hydrophobic peptide on the surface of ^68^Ga core-doped nanoparticles has been established to evaluate the acute and chronic inflammation by labeling neutrophils (Pellico et al., 2017). These radiolabeled NPs show huge potential application for real-time and rapid monitoring the development of sepsis.

Nanomaterials can be engineered through surface modification to specifically target sepsis-induced certain cells or tissues, thereby enhancing the sensitivity and specificity of these non-invasive imaging technologies. Besides, radiolabeled nanomaterials-based PET technology detects accurately the biological processes and detailed lesions of suffered organs, providing more comprehensive diagnostic information for sepsis management. Unlike traditional invasive diagnostic methods, novel nanomaterial-based diagnostic strategies show great potential in reducing the risk of sepsis-associated complications. Additionally, by determining the exact location, microstructure, metabolic status and severity of the suffered organs in sepsis, the combination of different nanomaterial-based imaging technologies provide great potential for future patients, enabling accurate identification and continuous monitoring of sepsis.

## 5 Treatment for sepsis

Although the understanding of sepsis has deepened, the guidelines for sepsis diagnosis and treatment worldwide are still being updated to cope with drug resistance problems and improve therapeutic treatment. It is worth noting that sepsis management is complicated and challenged (Alhazzani et al., 2020; Molnár et al., 2016; Rhodes et al., 2017), and that the evolution of pathogenic microorganisms, the complex pathophysiological mechanisms and multi-drug application make the situation worse. At present, guidelines emphasize the role of immediate antibiotic administration and fluid resuscitation in the early stage of sepsis treatment. Regrettably, the supportive therapy and timely administration of antibiotic therapy have been found to have little or no impact on lowering the of mortality patients (Busani et al., 2017). The bolus administration of fluid not only decreases arterial elasticity, but might also lead to major vasodilation and hyper-dynamic condition (Brown and Semler, 2019). On the other hand, excessive fluid administration was linked with tissue and organ failure, and ultimately death. Due to the ever-evolving increase in drug-resistant pathogens and marked limitations in the development of novel antibiotics, the research focus on the sepsis management and treatment has shifted accordingly. Recently, there are some urgent problems need to be solved, such as MDR, poor bioavailability, efficacy, targeting and selectivity of antibiotic (Truong et al., 2023). In this section, several different types of nanomaterials applied in the anti-septic nanomedicines will be analyzed and discussed, as well as their potential mechanisms.

### 5.1 Nanomaterials in antibiotic therapy

The advantage of nanomaterials-based antibiotic therapy in treating sepsis depends on the development of nanotechnology, achieving improved physicochemical properties of drugs, targeted and controlled antibiotic delivery, and appropriate pharmacokinetic profile (Liu et al., 2019; Gupta et al., 2019). Au NPs with β-lactam antibiotic-metallopolymers system displayed enhanced antibacterial effect, in which Au NPs served as excellent carriers for antibiotics. On the one hand, cationic metallopolymers insert into bacterial membranes via their hydrophobic groups, and the antibiotic-metallopolymers bioconjugates protected against the hydrolysis by the bacterial β-lactamase enzyme, thus regaining the activity of conventional antibiotics. On the other hand, the inherent advantages of Au NPs further enhanced the concentration of antibiotics in pathogenic bacteria, including small size for high permeability, high surface area for high loading capacity, surface modification for high targeting, *etc* (Yang et al., 2019). Shaker et al. demonstrated that grafting carbapenem on Au NPs increased the antibacterial efficacy of carbapenem against clinical resistant Gram-negative bacteria *in vitro*, suggesting that Au NPs could be a superior delivery vehicle for antibiotic, especially in the MDR infections (Shaker and Shaaban, 2017).

The liposomal formulation of amphotericin B, an antifungal agent used to treat fungal infections and fungal sepsis (Mitchell et al., 2021; Anselmo and Mitragotri, 2019; Stone et al., 2016), encapsulates the active ingredient amphotericin B within the bilayer of small (∼80 nm median size) unilamellar liposomes. AmBisome, marketed as Amphotericin B liposomes by Gilead Sciences, is as efficacious as the traditional version but can be administered at significantly higher doses due to its greater tolerability. First commercially available in 1997, AmBisome received FDA approval and is now accessible in over 58 countries worldwide, recognized as a safe and potent therapy for severe systemic fungal infections.

Dendrimers are highly branched and symmetrical macromolecules with multiple branches emanating from a central core (Gillies and Fréchet, 2005). Dendrimers can encapsulate hydrophilic and hydrophobic drugs and can be functionalized with targeting moieties (Choi et al., 2013). Ji et al., prepared the optimized telodendrimer (TD) nanocarrier for encapsulating monomeric Amphotericin B (AmB) to fine-tune the aggregation status of AmB, thus enhancing the antifungal activity and reducing the cytotoxicity (Ji et al., 2023). With the hydrophilic exteriors and interiors, dendritic NPs maintain the property of unimolecular micelle to engineer precisely core structures for AmB encapsulation (Abbasi et al., 2014).

Characterized with controlled sizes and surface properties (Bernardos et al., 2019), silica NPs usually possesses high loading capacity for antibiotics, as well as protection against degradation of the agent. Additionally, Alavi et al. evaluated the antibacterial effect of the loading of nafcillin (NF) into a (PEG) ylated liposome (PEG-Lip-NF), which was a promising strategy against methicillin-susceptible *Staphylococcus aureus* (MSSA) bacteremia and biofilm-associated infections (Alavi et al., 2022). Nafcillin encapsulated by PEG-coated liposome exhibited more potent anti-MSSA activity in the MIC and MBIC50 tests, decreased virulence, and increased survival rate induced by MSSA bacteremia, compared with NF-loaded liposome or NF alone. Surprisingly, PEG-Lip-NF decreased the NF-provoked nephrotoxicity in the MSSA infected animals (Alavi et al., 2022), suggesting that PEGylated liposome is a promising nano-carrier in MSSA infection.

**FIGURE 3:**
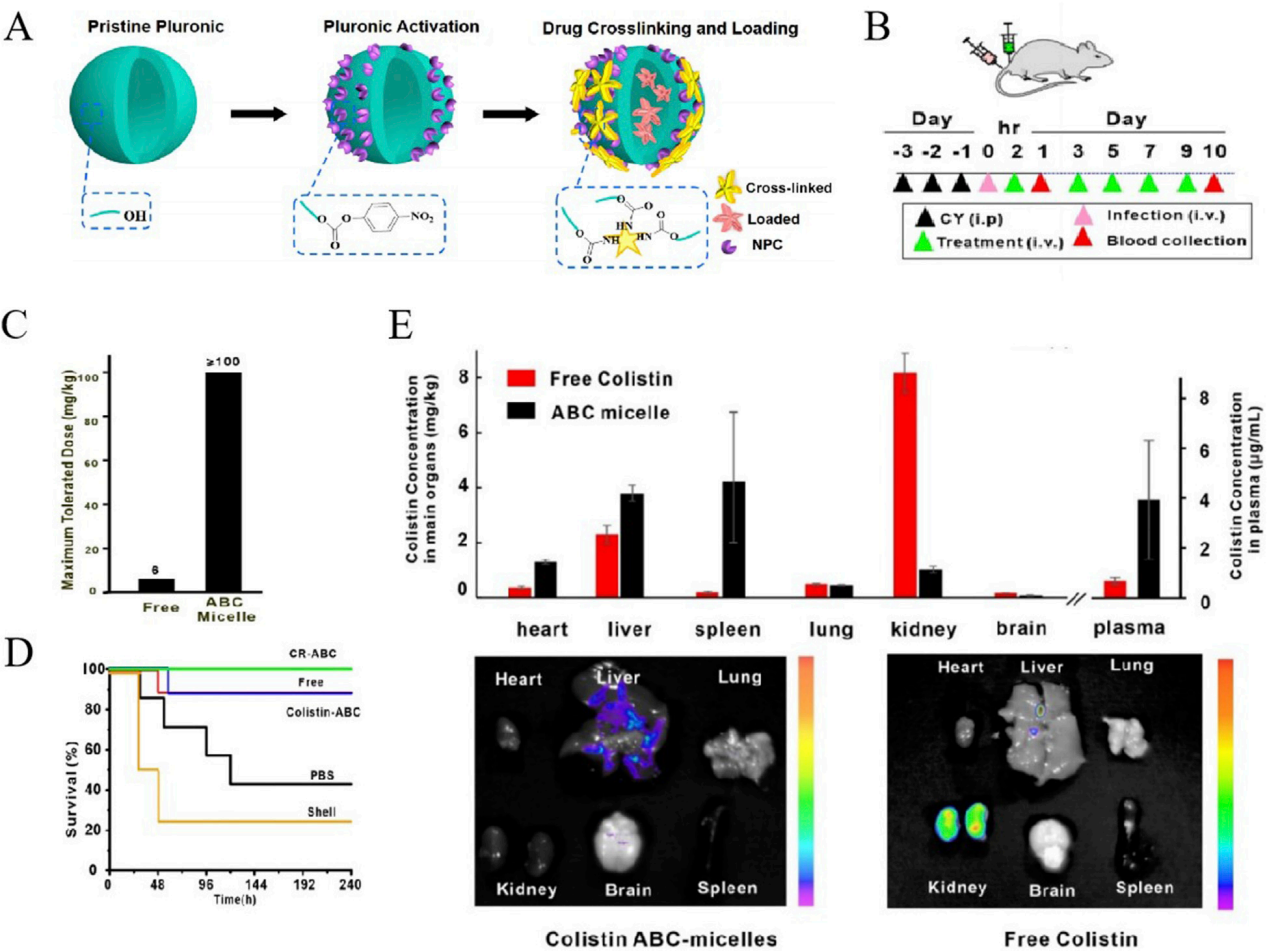
**(A)** Schematic of the formation of colistin ABC-micelles. **(B)** Treatment of sepsis by colistin-rifampicin ABC-micelles Schematic of experimental design. **(C)** MTD of free colistin and colistin ABC-micelles. **(D)** Survival rates for different groups. n = 7–8 per group. **(E)** Biodistribution of free colistin and colistin ABC-micelles 24 h after injection. Independent mice for halflife and biodistribution. Representative fluorescent images of main organs including heart, liver, spleen, lung, kidney, and brain 24 h after injection of colistin ABC-micelles and free colistin (Yang et al., 2021). Reproduced with permission from ACS publications Ltd. Copy-right 2018, American Chemical Society.

To reduce the toxicity of colistin, a novel colistin formulation was designed, which was prepared with colistin antibiotic cross-linked micelles (ABC micelles) in aqueous solution by a novel one-pot method (Figure 3A). As we known, colistin characterized by its amine-rich structure and a solitary hydrophobic tail, possesses an exceptional capacity to bind to and destabilize the outer membrane of bacteria, destroying the integrity of membrane to release the components of infected bacteria, ultimately contributing to bacterial death. However, its amine-rich composition also leaded to nephrotoxicity and neurotoxicity, presenting a significant dose-limiting issue. In the study conducted by Yang et al., the terminal hydroxyl group of Pluronic F127 was activated with p-nitrophenyl chloroformate (NPC) to facilitate a reaction with the amine groups of colistin (Yang et al., 2021). Consequently, a portion of colistin was cross-linked with micelles, which not only reduced the amine-rich toxicity but also encapsulated the remaining of free colistin. Thus, compared with the existing conventional therapies, nanomaterial-based strategies exhibited same safety and higher efficacy. At temperatures around 4°C, these ABC micelles, unreacted Pluronic, and loosely bound surfactants dissociate into Unimers. Subsequently, the free colistin can be purified through low-temperature centrifugal filtration, yielding purified ABC micelles with a high concentration. This innovative approach has resulted in ABC micelles that not only substantially boost the encapsulation of antibiotics but also notably diminish systemic toxicity in mice. The mice were challenged with a multidrug-resistant strain of *E. coli* and subjected to various treatments, as illustrated in Figure 3B. The toxicity assessment, as presented in Figure 3C, revealed that the ABC micelles were well-tolerated, demonstrating a remarkable increase in the maximum tolerated dose (MTD) by at least 16-fold when compared to free colistin. Most notably, the group treated with colistin-ABC micelles experienced a significant enhancement in survival rates, comparing with the other treatment groups (Figure 3D). Furthermore, the biodistribution of ABC micelles was characterized by a preferential accumulation in the liver and spleen, with a significant reduction in kidney uptake, as shown in Figure 3E (Yang et al., 2021).

The deployment of these NPs in antibiotic delivery represents a promising frontier, offering distinctive attribution and functionalities. These have the potential to enhance drug efficacy, refine targeted therapies, reduce the toxicity of antibiotic, and retune the drug profiles of absorption, distribution, metabolism and excretion (ADME). Nonetheless, it is imperative to recognize that the ongoing research is dedicated to thoroughly assessing their safety profiles in the long-term implications of biomedical applications.

### 5.2 Nanomaterials of antimicrobial peptides

Antimicrobial peptides (AMPs) are an important part of the innate immunity of host, and are regarded as natural antibiotics synthesized by various organisms, including mammals, plants, protozoa, fungi, and bacteria. AMPs have amphipathic or cationic structural compositions and display a wide range of antimicrobial activity, targeting both Gram-positive and Gram-negative bacteria, fungi, viruses, and protozoa (Huan et al., 2020). Compared with traditional antibiotics, AMPs exhibit stronger immunomodulatory ability, contributing to the elevated innate immunity and acquired immunity by modulating various immune cells. Owing to the unique bacteria-lysing mechanism and anti-MDR property, AMPs has gained much attention in anti-infection field (Tan et al., 2022). However, the immature stability, uncertain safety profile, higher nephrotoxicity and neurotoxicity of AMPs hindered the research and potential application for sepsis therapy.

**FIGURE 4:**
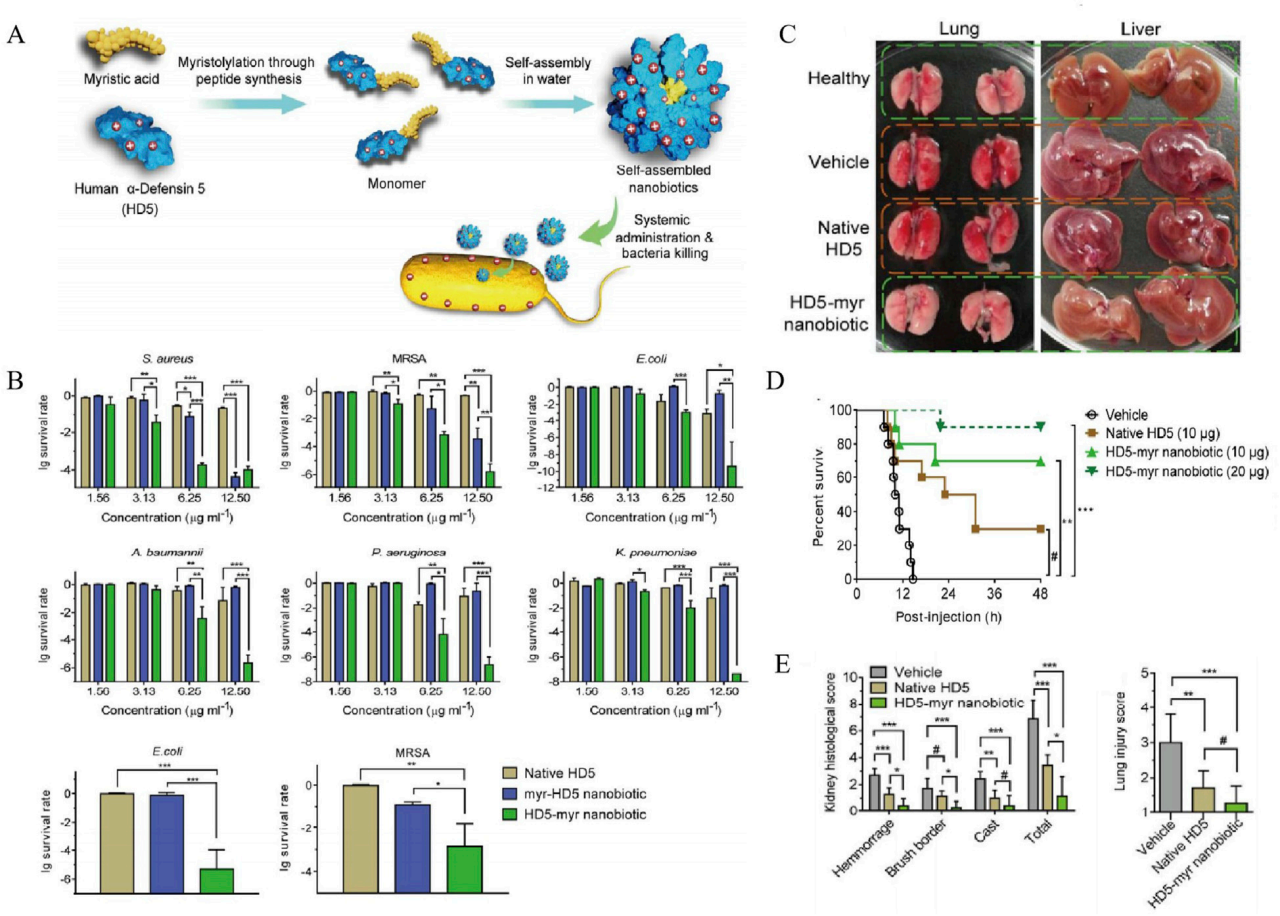
**(A)** Rational engineering of human alpha-defensin 5 (HD5) through peptide myristoylation at the N- or C-terminus and sequential nanoassembly of myristoylated HD5 for generating nanobiotics. **(B)** Antimicrobial activity of HD5 and its myristoylated counterparts, myr-HD5 nanobiotic (NB) and HD5-myr NB. Dose-dependent survival of *Staphylococcus aureus*, methicillin-resistant *Staphylococcus aureus* (MRSA), *Escherichia coli*, **(A)** baumannii, *P. aeruginosa*, and *K. pneumoniae* treated with HD5 and NBs. **(C)** Organs of normal mice and of diseased mice treatment with vehicle, HD5 or the HD5-myr NB. **(D)** Kaplan-Meier survival analysis of mice treatment with vehicle, HD5, the HD5-myr nanobiotic (NB), or the HD5-myr NB. **(E)** The severity of injury in the kidney and lung (Lei et al., 2018). Reproduced with permission from ACS publications Ltd. Copy-right 2018, American Chemical Society.

To solve these problems, a self-assembled NPs loaded with of human alpha-defensin 5 (HD5), a natural AMP secreted by intestinal Paneth cells, was designed and prepared (Figure 4A), with improved stability, bio-safety, and antibacterial potency (Lei et al., 2018). The nanobiotic-based formulations for drug-specific targeting exhibited significantly enhanced broad-spectrum bactericidal activity *in vitro* against various pathogens, including *S. aureus*, MRSA, *Escherichia coli* (*E. coli*), *etc.*, compared to the free HD5 (Figure 4B). The analysis of organ injury scores, as presented in Figure 4C, fully supports these observations, highlighting the HD5-myr nanobiotic’s capacity to safeguard mice against sepsis. As illustrated by Figures 4D,E, HD5-myr nanobiotic treatment significantly reduces the mortality and organ damage in MSRA and E. coli-induced septic murine model, favoring the self-assembled HD5-myr nanobiotic in the application prospect for bacterial sepsis management.

In addition, Tan et al. synthesized antibacterial peptide (KR-12: KRIVKRIKKWLR)-grafted amphiphilic block copolymer and biotin grafted block copolymer, which were co-assembled in an aqueous solution to produce the AMPNP (Tan et al., 2022). In this work, the *in vitro* predominant antibacterial activity of the prepared NPs was confirmed, including *E. coli*, *S. aureus*, and MRSA. Moreover, in a septic mice model, both nanosystems showed a more pronounced reduction in serum cytokines (IL-1β, TNF-α, and IL-6) and inflammatory infiltration in target tissues compared to the uncoated AMPNPs. Saúde et al. have encapsulated the antimicrobial peptide Clavanin A in a polymeric matrix for bacterial sepsis control, aiming to improve its stability and therapeutic efficacy (Saúde et al., 2014). A polymicrobial sepsis model of mice was adopted to evaluate the *in vivo* efficacy of the nanoantibiotic Clavanin A, which showed a 100% survival rate under a sub-lethal dose of bacteria and a 40% survival rate with a lethal inoculum, along with improved antimicrobial activity and druggability.

The micellar form of CG3R6-TAT peptide, presented as nanodots, has demonstrated efficacy in curing sepsis-induced meningitis in the central nervous system, as reported by Martin et al. (2015). By modifying the antimicrobial peptide HHC36 (KRWWKWWRR) with the aggregation-induced emission (AIE) molecule 2-(2-hydroxyphenyl)benzothiazole (HBT), an AIE probe named AMP-2HBT was prepared. This probe not only retains the antimicrobial activity of the HHC36 peptide but also allows for real-time observation of the binding process between the antimicrobial peptide and bacteria, thus revealing the mechanism by which the antimicrobial peptide HHC36 kills bacteria by disrupting the structure of bacterial membranes (Chen et al., 2018). Additionally, polyprodrug antimicrobials, such as triclosan and methacrylate, have been synthesized and have shown significant therapeutic efficacy against methicillin-resistant *S. aureus*, as documented by Schuerholz et al. (2012).

There is no doubt that nanomedicines loaded with antimicrobial peptides have great potential in the sepsis management, but there are still many problems to be solved from basic to clinical research. Indeed, many clinical trials nanomedicines of antimicrobial peptides in the treatment of sepsis are still in their infancy, and a large number of relevant clinical data need to be collected for the specific therapeutic dosage, therapeutic time window and the administration routes. What’s more, much more attention should be paid to the antimicrobial peptides resistance in the future research.

### 5.3 Cell membrane-coated biomimetic nanoparticles

Cell membrane-coated nanoparticles (MNPs) are the novel biomimetic NPs that are created by the surface of synthetic NPs with natural cell membranes. MNPs retain both the physicochemical properties of synthetic nanomaterials and the inherent functions and features of the origin cells, such as “self-labeling” and the interaction with immune system. Therefore, MNPs possess better biocompatibility and weaker immunogenicity, enabling them to evade the immune clearance. Unlike other nanoparticles, the targeted delivery of MNPs is achieved by the recognition mechanism of engineered membrane proteins on MNPs to ligands, which allows for more efficient delivery of encapsulated drug (Sushnitha et al., 2020; Oroojalian et al., 2021). Herein, we summarized the progress of three main categories of MNPs achieved in sepsis treatment.

#### 5.3.1 Platelet membrane-coated nanoparticles (PNPs)

**FIGURE 5:**
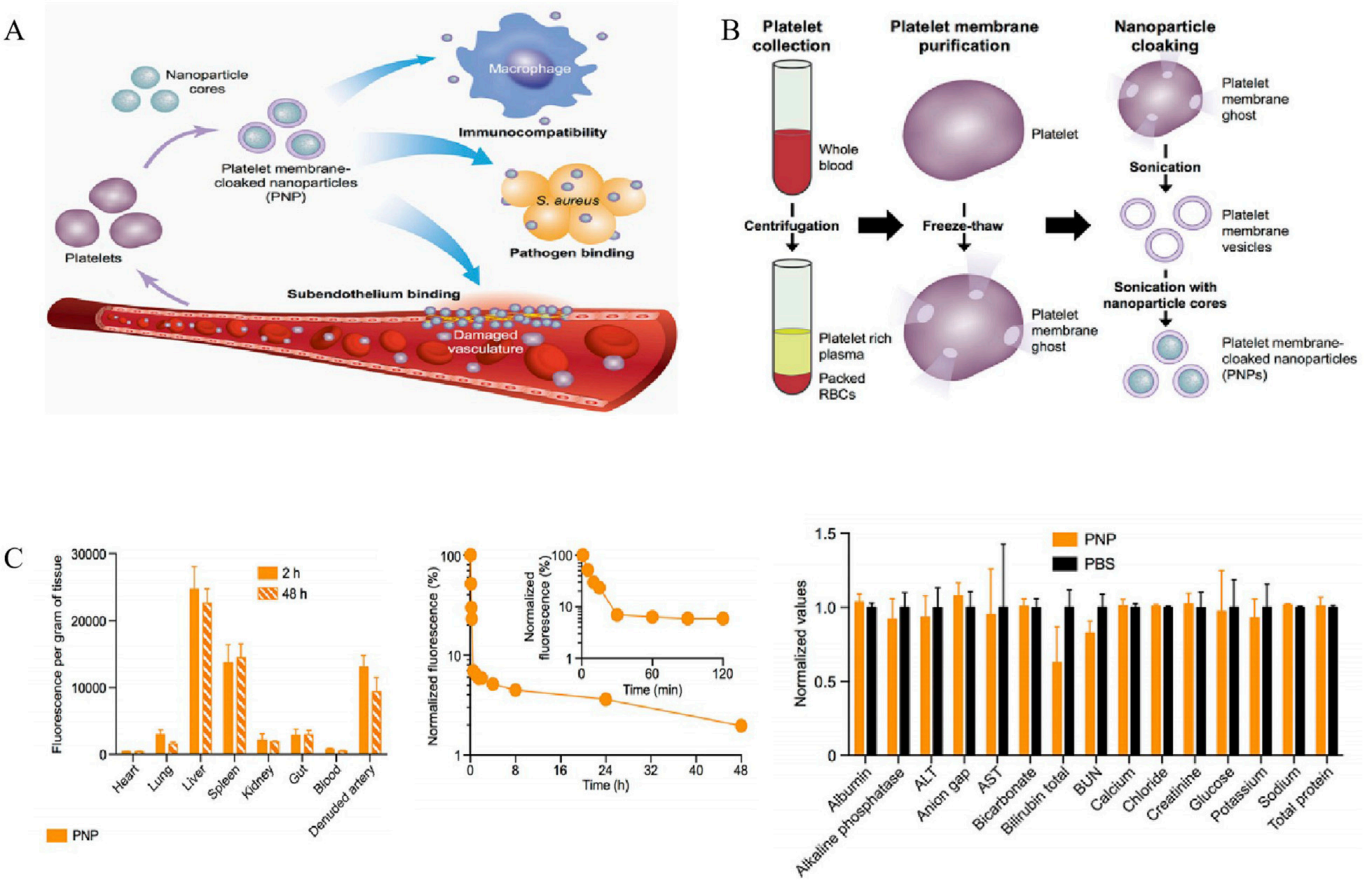
**(A)** Poly (lactic-co-glycolic acid) (PLGA) nanoparticles are enclosed entirely in plasma membrane derived from human platelets. The resulting particles possess platelet-mimicking properties for immunocompatibility, subendothelium binding, and pathogen adhesion. **(B)** Schematic depicting the process of preparing PNPs. **(C)** Pharmacokinetics, biodistribution, and safety of PNPs. All bars and markers represent means ± SD (Hu et al., 2015). Copyright Year 2015 Springer Nature.

Platelets (PLs) play a crucial role in the host immune defense against infection through various mechanisms, such as endocytosis of pathogen and elimination of bacterial toxins. Furthermore, it has been well established that the activation of platelets is involved in the inflammation and coagulation issues, which contributing to the development of sepsis (Li T. et al., 2020). It has been documented that 35%–59% patients with sepsis developed thrombocytopenia, which is a risk causes of mortality of sepsis. Considering that PLs could adhere to bacteria and bacteria-secreted toxins (Shannon, 2015), these interactions potentially enhance their capacity for active targeting and reduce bacterial virulence. In 2015, Hu et al. discovered a platelet membrane-coated poly (lactic-co-glycolic acid) (PLGA) NPs (Figures 5A, B) with superior bactericidal effect. These NPs possessed platelet-mimicking properties, including excellent immune compatibility, pathogen adhesion, and subendothelial binding. Notably, compared to the free vancomycin (Vanc) at six times the dosage, Vanc loaded-PNPs (PNPs-Vanc) showed significantly higher antimicrobial properties in the blood and the vulnerable organs (heart, lung, kidney, livers and spleen) (Figure 5C).

#### 5.3.2 Red blood cell membranes-coated nanoparticles (RBC NPs)

RBCs are naturally occurring, long-circulating carriers that can remain viable in human bodies for up to 120 days. Naturally capable of binding to toxins, RBC membranes can intercept and neutralize bacterial toxins. Hu et al. discovered a detoxification effect of RBC membrane-coated PLGA NPs (denoted as nanosponges) in staphylococcal alpha-haemolysin (α-toxin)-induced sepsis model (Hu et al., 2013). RBC vesicles could effectively retain large amounts of α-toxin and increase its hemolytic action, compared to the PLGA NPs and liposomes. Additionally, *in vivo* experiments suggested that RBC NPs decreased the acute mortality in the lethal α-toxin-challenged mice (Hu et al., 2013). To further enhance the biodistribution and detoxification effect of PLGA nanoparticles, an innovative design combining physical and chemical biomimicry of biodegradable PLGA nanoparticles was developed (Ben-Akiva et al., 2020). This RBC NPs platform significantly elevated the survival rate of septic mice, which was attributed to the synergistic effect of the anisotropic shape and membrane coating via protection against macrophage uptake and reduction of nanoparticle clearance. In general, the anisotropic RBC NPs offer alternative optimal particle formulations for septic detoxification strategies.

#### 5.3.3 Macrophage cell membrane-camouflaged nanoparticles (MΦ-NPs)

**FIGURE 6:**
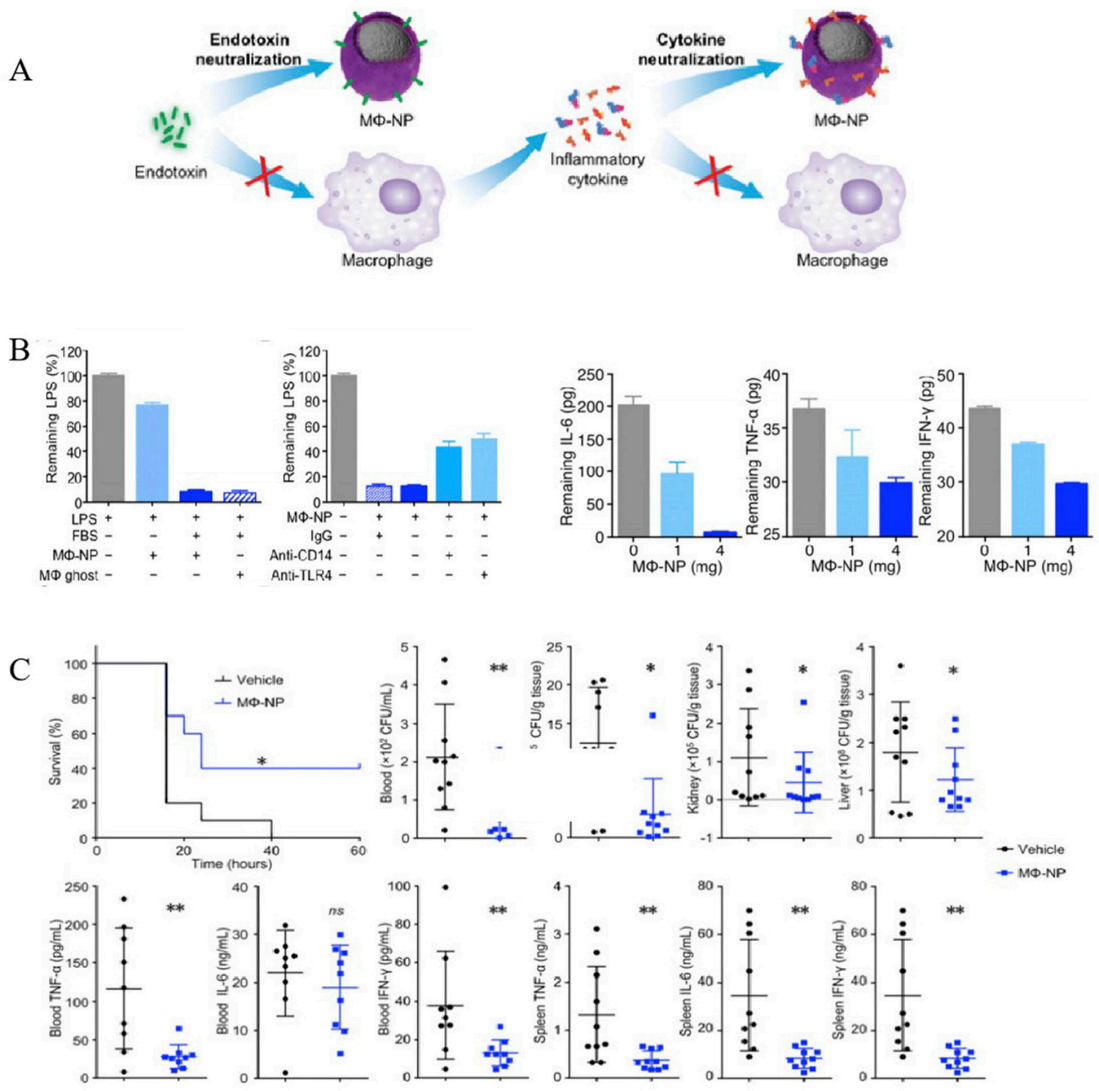
MΦ-NPs concurrently absorb endotoxin and proinflammatory cytokines to treat sepsis. **(A)** Schematic illustration of MΦ-NPs neutralizing endotoxin and proinflammatory cytokines. **(B)** Left: the capacity of MΦ-NPs to remove LPS in the presence and absence of LPS binding protein. Right: non-specific IgG and antibodies that block CD14 and TLR4. Removal of indicated proinflammatory cytokines with MΦ-NPs. **(C)** Evaluation of the therapeutic efficacy of MΦ-NPs on mice by survival rate of mice, bacteria counts, and levels of proinflammatory cytokines (*P ≤ 0.05, **P ≤ 0.01) (Thamphiwatanaa et al., 2017). Copyright Year 2017 National Academy Sciences.

Macrophages (MΦs) play an important role in the development of sepsis, participating in innate immunity and adaptive immunity through polarization, autophagy, and regulation of the inflammatory response. In the early stage of sepsis, macrophages tend to polarize towards M1 phenotype to combat infection, while they polarize towards M2 phenotype in the late stage due to immunosuppression and infection aggravation caused by immune failure. Owing to their propensity for inflammation and infection sites, MΦ-NPs have gained much more attention worldwide in the treatment of inflammation and inflammation-related diseases, including sepsis (Lee et al., 2022). Thamphiwatana et al. first developed macrophage biomimetic nanoparticles by fusing membrane vesicles derived from MΦs onto PLGA cores, whose outside surface possesses the same antigenicity as macrophages (Thamphiwatanaa et al., 2017) (Figure 6A). Acting as a bait for macrophages, these nanoparticles can bind to and neutralize endotoxins that trigger immune activation, as well as pro-inflammatory cytokines that trigger pathological “cytokine storms”. As shown in Figure 6B, MΦ NPs can effectively sequester various types of proinflammatory cytokines in a concentration-dependent manner. Moreover, *in vitro* assays indicated that MΦ-NPs could adhere to LPS and remove proinflammatory cytokines, such as IL-6, TNF, and IFN-γ. The bacterial levels in the blood, spleen, kidney and liver of the mice treated with MΦ-NPs were significantly lower than those in the control group (Figure 6C). Consequently, these results manifested that MΦ-NPs might be a biomimetic detoxification strategy of sepsis management with a significant inhibitory activity of proinflammatory cytokines in both bloodstream and organs (spleen).

In general, these biomimetic nanoparticles (NPs) have shown enhanced efficacy in immunomodulatory applications, such as combating infections, managing inflammation, reversing immunosuppression, and facilitating drug-targeting delivery. This robust performance strongly suggests their potential in sepsis treatment, as highlighted by Wang et al. (2022). Although the majority of these studies are in their nascent stages and no distinct biomimetic NPs have reached the market yet, they have already begun to overcome several challenges associated with traditional therapeutics—like limited bioavailability and a lack of precise targeting—and with bare nanoparticles, such as brief retention times and potential immunogenicity. Consequently, biomimetic nanomedicines are poised to significantly advance the development of innovative sepsis treatments, thus improving patient outcomes.

Although the aforementioned results are promising, there remains a significant journey ahead before these biomimetic NPs can be effectively utilized in sepsis treatment. The first technological challenge is ensuring the uniformity and the integrity of their coating membranes, and maintaining the activity of membrane protein, to guarantee the quality control of biomimetic NPs, as noted by Li M. et al. (2020). Secondly, it has been demonstrated that different types of nanoparticles disrupt the reproductive system via cytotoxicity and oxidative stress, including carbon nanomaterials, dendritic polymers, quantum dots, silica, and gold nanoparticles. Therefore, the reproductive toxicity and long-term application of biomimetic NPs needs to be studied more comprehensively, as discussed by Ismail et al. (2022). Thirdly, although the current preparation of biomimetic membranes onto NPs has been achieved in laboratory settings, the techniques for scaling up these processes need further development to meet the demands of large-scale clinical applications. Last but not the least, functional diversity of biomimetic nanoparticles might be limited due to a single or conventional type cell membrane as the coating material. Therefore, the nanoparticles coated by hybrid membrane may be one of the future research directions.

### 5.4 Nanomaterials targeting macrophages for sepsis management

Macrophages are phagocytes in the innate immunity dedicated to the recognition of foreign substances, including NPs, with which an immune response subsequently occurs. Various design strategies, such as surface functionalization, have been implemented to manipulate the recognition and engulfment of NPs by monocytes/macrophages, regulating their function in sepsis, and compensating for the shortcomings of anti-infective conventional strategies (van der Poll et al., 2017; Locati et al., 2020). Considering the critical role of prolonged oxidative stress in sepsis-associated injuries, a novel H_2_O_2_ scavenger platform was developed by assembling bovine serum albumin/manganese oxide (BSA/MnO2) nanoparticle onto mannosylated disulfide cross-linked polyethylenimine (ssPEI) (mSP) (Rajendrakumar et al., 2018). This synthesized nanoassemblies improved the stability of MnO2, specifically inhibited the production of H_2_O_2_-mediated free radicals, rather than other superoxide free radicles. The highly stable mSPAM nanoassembly inhibited HIF1α expression by clearing H_2_O_2_ in LPS-induced macrophages, decreased serum TNF-α and IL-6 levels, and reduced infiltration of neutrophils and other white blood cells in endotoxemia models, thereby avoiding subsequent organ damage induced by inflammatory macrophages. More recently, a nanomedicine for conditioning macrophages was designed and prepared by coating the albumin-methyl palmitate with spherical nanoparticles (namely, spherical methyl palmitate nanoparticles, MPN), which can effectively transport natural fatty acids and temporarily inhibit the uptake of nanomaterials by resident macrophages (Palomba et al., 2021), providing a new idea for macrophages-targeting sepsis management.

The intricate pathophysiology of sepsis alters the phenotype and function of macrophages, leading to a state of macrophage exhaustion. Currently, there is a dearth of drugs that effectively targeting macrophage function and a lack of robust methods for detecting macrophage activity. Encapsulated and sequestered the active pharmaceutical ingredients, nanomaterials emerge as promising candidate materials for macrophage targeting in sepsis, which enhance targeted delivery to macrophages over time and improve the biodistribution and bioavailability of drugs.

### 5.5 Natural products loaded nanomaterials for sepsis

Natural products have long been used to prevent and treat various diseases, including inflammatory and immune-related disease (Gu et al., 2022). Due to their excellent anti-inflammatory, antibacterial and immunoregulatory potency in sepsis as well as the low cost, they have garnered considerable concern (Song et al., 2023). However, the low solubility, hydrophobicity and low bioavailability have limited their broader application of them in clinical (Huang et al., 2022). In recent years, researchers have paid more and more attentions to using nanomaterials load natural products, reducing the side effect of them and control the drug release for nanosystems. Herein, we summarize natural products that have been loaded into nanosystems for sepsis treatment in Table 1, such as curcumin (Cur), tea polyphenols, resveratrol and so on. As shown in Table 1, Wang et al. successfully prepared and evaluated Cur-loaded solid lipid nanoparticles (Cur-SLNs) for sepsis (Wang et al., 2015). *In vitro* experiments indicated that Cur-SLNs can effectively reduce levels of IL-1β, TLR4, TLR2, and TNF-α. Meanwhile, the results showed suppression of NF-κB activation and IκBα degradation levels. Similar results were observed in *vivo* experiments*.* Altogether*,* they proposed that Cur-SLNs could serve as a promising therapeutic drug delivery system for LPS-induced sepsis through the TLR2/4-NF-κB signaling pathway.

**TABLE 1:** Summary of natural products loaded nanosystems for sepsis treatment.

Nanosystem types	Natural products	Key evaluations	Mechanisms of action	Ref
Cur-loaded Solid lipid nanoparticles (Cur-SLNs)	Curcumin	DLS TEM *In vitro* efficacy *In vivo* efficacy	Active the NF-kB signaling pathway in LPS-induced murine macrophage cells	Wang et al. (2015)
Chitosan loaded with CeO_2_ and Cur (Cur@Ce/OCS NPs)	Curcumin	*In vitro* efficacy *In vivo* efficacy	Reduce bacterial burden; decrease inflammatory responses in sepsis model	Teng et al. (2023)
Cip·HCl/Cur@Lip-γ3	Curcumin	*In vitro* efficacy *In vivo* efficacy	Kill bacteria and regulate macrophages to relieve the infected microenvironment; provide antibacterial–anti-inflammatory therapy for synergetic	Zhang et al. (2023)
Tea polyphenols nanoparticles (TPNs)	Tea Polyphenols	DLS TEM SEM *In vitro* efficacy *In vivo* efficacy	By inhibiting gasdermin D (GSDMD) mediate pyroptosis blockage	Chen et al. (2022)
Resveratrol loaded silver nanoparticles (AgNPs + RV)	Resveratrol	EDX STEM *In vitro* efficacy *In vivo* efficacy	Promote SIRT1 activation	Üstündağ et al. (2023)
Nanoemulsions (NE) loaded with Tea tree oil (TTO):NE-TTO Nanocapsules (NC) loaded withTea tree oil (TTO):NC-TTO	Melaleuca alternifolia	TEM Cytotoxicity assay *In vitro* efficacy *In vivo* efficacy	Increase group thiols in the kidney and lungs; reduce the bacterial growth in the peritoneal lavage	Battisti et al. (2024)
Peel-derived loaded silver NPs (PGNPs)	Pomegranae peel extracts	*In vitro* efficacy *In vivo* efficacy	Increase levels of glutathione; reduced concentrations of nitric oxide controlled apoptosis-related genes	Marey et al. (2024)

However, the majority of nanomedicines loaded with active natural compounds are still in the preclinical stage, and the release mechanism and its clinical therapeutic effect need to be further clarified.

### 5.6 Other nanoformulations for sepsis management

Photothermal antimicrobial therapy represents an innovative treatment approach that utilizes light-induced heat to eradicate or inhibit the growth of kinds of pathogenic pathogens. These alternative options comprise photo-sensitive NPs and specialized dyes, *etc.*, which are adept at absorbing specific light wavelengths and transforming that energy into thermal heat. In the realm of nanotechnology, nanozymes are nanomaterials that possess the intrinsic enzyme-like activities (Huang et al., 2019). As synthetic catalysts, nanozymes stand out as a potential substitute for natural enzymes across a variety of applications, notably in their capacity to eliminate bacterial infections (Zhang et al., 2021).

## 6 Future trends and challenges

Nanotechnology-based strategies are emerging as a promising frontier in sepsis treatment, offering a multifaceted approach that includes targeted drug delivery, enhanced antibiotic efficacy, anti-inflammatory action, and immunomodulatory capabilities (Hu et al., 2023; Qian et al., 2023). Preclinical research has yielded encouraging outcomes regarding the application of nanotechnology in combating sepsis. For instance, dexamethasone-grafed polymeric NPs have been observed to lower proinflammatory cytokine levels in a LPS-provoked septic murine model (Zhai et al., 2022). Furthermore, liposomes encapsulating Vanc have demonstrated a positive impact on survival rates and a significant reduction on bacterial load in a MRSA-triggered septic model (Nwabuife et al., 2021). Additionally, dendrimers equipped with TLR agonists have been found to boost the phagocytic capabilities of macrophages and enhance survival rates of E. coli-induced septic mouse (Jackson Hoffman et al., 2023).

In conclusion, the potential advantages of therapeutic NPs include: (1) Ability to improve the pharmaceutical and pharmacological properties of drugs to lower the minimum effective dose of the drug. (2) Enhancement of therapeutic efficacy by drug-targeting delivery to reduce the side effects, including the toxicity of the drug itself and the cumulative toxicity in specific organs. (3) Delivery of drugs directly to the infected sites. (4) Playing a synergistic therapeutic role with potentially different physicochemical properties. (5) Real-time monitoring of therapeutic efficacy by integrating the noninvasive imaging technology and high-efficiency therapeutic nanomedicines.

However, several challenges need to be addressed before these nanomaterials can be effectively integrated into clinical practice. One challenge is evaluating the safety profile of nanoparticle-based approaches, particularly in terms of long-term toxicity and potential for accumulation in the body. Another challenge is that regulatory approval for nanotechnology-based therapies can be difficult due to the complex nature of nanomedicines and the lack of standardized manufacturing processes. Moreover, there are also ethical considerations to be addressed, especially in the context of preclinical and clinical studies involving nanomaterials. Currently, the application of cost-effectiveness studies within the realm of nanomedicines remains in its nascent stages (Bosetti et al., 2013).

In this article, we will explore the safety concerns, regulatory laws, ethical considerations and cost-effective for nanomedicines. A primary concern revolves around the safety of nanomedicines. Comprehensive safety assessments are essential, including evaluations of acute toxicity, reproductive toxicity, immunogenicity, and biodistribution, to understand their potential impact on human health fully. As with any innovative therapeutic approach, thorough safety evaluations are paramount to ensure a secure and efficacious transition of nanomedicines from the laboratory to clinical settings. In addition, when producing nanomedicines on an industrial scale, it is necessary to consider how to scale up the laboratory-scale preparation techniques to an industrial scale, which is responsible for the consistency and quality stability of nanomedicines. The development of novel preparation procedures and the amplification of processes are beneficial for the elevation of production efficiency, such as microfluidic technology. The path from laboratory development to clinical application requires strict adherence to regulatory frameworks, encompassing preclinical and clinical trials, as well as guidelines for manufacture, labeling and approval. Moreover, this rigorous process is vital to ensure that nanomedicines not only meet the standards of efficacy and safety but also align with the legal and ethical requirements for patient care. Besides, offering the most cost-effective solutions for specific health outcomes, interventions that are economically efficient provide excellent value to society.

At present, the emerging technologies and innovative approaches are important in nanomaterials-based sepsis management, mainly including nanomedicines drug delivery systems, responsive nanomedicines drug delivery systems, membrane-camouflaged biomimetic NPs, detoxification NPs, O_2_^−^ scavenger NPs, immunomodulatory NPs, and the application of NPs in vaccine development, among others. We look forward to the emergence of more methods and technologies for treating sepsis in the future.
